# Accuracy of Urine Kidney Injury Molecule-1 in Predicting Acute Kidney Injury in Children; a Systematic Review and Meta-Analysis

**Published:** 2020-04-05

**Authors:** Mojtaba Fazel, Arash Sarveazad, Kosar Mohamed Ali, Mahmoud Yousefifard, Mostafa Hosseini

**Affiliations:** 1 Pediatric Chronic Kidney Disease Research Center, Tehran University of Medical Sciences, Tehran, Iran.; 2 Department of Pediatrics, Valiasr Hospital, Imam Khomeini Medical Complex, Tehran University of Medical Sciences, Tehran, Iran.; 3 Colorectal Research Center, Iran University of Medical Sciences, Tehran, Iran.; 4 Nursing Care Research Center, Iran University of Medical Sciences, Tehran, Iran.; 5 College of medicine, University of Sulaimani, Sulaimani, Iraq.; 6 Physiology Research Center, Iran University of Medical Sciences, Tehran, Iran.; 7 Department of Epidemiology and Biostatistics, School of Public Health, Tehran University of Medical Sciences, Tehran, Iran.

**Keywords:** Acute Kidney Injury, Renal Insufficiency, HAVCR1 protein, human, Hepatitis A Virus Cellular Receptor 1

## Abstract

**Introduction:**

There is considerable controversy on the accuracy of Kidney Injury Molecule-1 (KIM-1) in prediction of acute kidney injury (AKI) in children. Therefore, the present study intends to provide a systematic review and meta-analysis of the value of this biomarker in predicting AKI in children.

**Methods:**

An extensive search was performed on the Medline, Embase, Scopus and Web of Science databases by the end of 2019. Cohort and case-control studies on children were included. Urinary KIM-1 levels were compared between AKI and non-AKI groups. Findings were reported as an overall standardized mean difference (SMD) with a 95% confidence interval (CI). Also, the overall area under the receiver operating characteristic (ROC) curve (AUC) of KIM-1 in predicting AKI in children was calculated.

**Results:**

Data from 13 articles were included. Urinary KIM-1 levels in children with stage 1 AKI were higher than the non-AKI group only when assessed within the first 12 hours after admission (SMD = 0.95; 95% CI: 0.07 to 1.84; p = 0.034). However, urinary KIM-1 levels in children with stage 2-3 AKI were significantly higher than non-AKI children (p <0.01) at all times. The AUC of urinary KIM-1 in predicting AKI in children was 0.69 (95% CI: 0.62 to 0.77).

**Conclusion:**

Based on the available evidence, KIM-1 seems to have moderate value in predicting AKI in children. Since previous meta-analyses have provided other urinary and serum biomarkers that have better discriminatory accuracy than KIM-1, so it had better not to use KIM-1 in predicting AKI in children.

## Introduction:

Acute kidney injury (AKI) is one of the major public health problems worldwide, with a high incidence and many new cases annually (1). There are many complications that can result from this condition, including metabolic acidosis, elevated blood potassium levels, uremia, and changes in fluid balance. Long-term complications of AKI also include cardiovascular disease, stroke, and heart failure.

Children with AKI mainly die from cardiovascular diseases and infections (2). Current research suggests that the use of preventive strategies and rapid diagnosis of AKI can lead to a significant reduction in the burden of AKI (3). Prompt diagnosis and treatment of the disease will enable slowing down the progression of the disease and prevent it from causing lasting complications, such as chronic kidney failure. Despite significant advances in medical knowledge, delayed identification of AKI can occur in some cases, and this may lead to persistent damage (4-6). Therefore, researchers are looking for diagnostic methods for early AKI identification.

In recent years, serum and urine biomarkers have been suggested as reliable methods for rapid diagnosis of renal diseases, they have been shown to have better prognostic value compared to other techniques (7-9). These factors include serum creatinine, cystatin C, neutrophil gelatinase-associated lipocalin (NGAL) protein, and Kidney Injury Molecule-1 (KIM-1) (10-12).

KIM-1 is a membrane protein, which is not detectable in serum/urine of healthy individuals. However, KIM-1 is widely expressed in proximal tubule cells after ischemia and toxic conditions, and has been reported to be an appropriate marker in the diagnosis of AKI (13, 14). Systematic reviews and meta-analyses of adult studies suggest that urinary KIM-1 levels can be an appropriate marker for early detection of AKI (14, 15).

As can be seen, these meta-analyses were mainly performed on adults, while the number of studies on children has increased in recent years. In addition, there is considerable controversy on the accuracy of KIM-1 in prediction of AKI in children. Therefore, the present study intends to provide a systematic review and meta-analysis of the value of this biomarker in predicting AKI in children.

## Methods:

**- Study design**

This meta-analysis was designed based on the guidelines for Meta-analysis of Epidemiology Statement, to evaluate the value of urinary KIM-1 level in predicting AKI in children (16).

**- Search strategy**

To achieve the objectives of the present study, extensive searches were conducted on Medline (via PubMed), Embase, Scopus, and Web of Science by the end of 2019. The search strategy was based on words related to KIM-1 and AKI. Then, by combining these words with appropriate tags in the databases, searches were performed and relevant articles were screened. To find additional articles or unpublished data, manual search was performed in the bibliography of relevant studies as well as search in Google and Google Scholar search engines. The search query used in Medline database is reported in appendix 1.

**- Selection criteria**

PICO was defined as follows: P: paediatric patients with AKI, I: urinary KIM-1, C: compare with non-AKI children, and outcome: discriminatory accuracy of KIM-1. In the present study, cohort and case-control studies on the accuracy of KIM-1 in predicting AKI in children were included. Studies were included if AKI was confirmed by a standard procedure and urine samples were obtained from all participants. Duplicate studies, review studies, studies without a non-AKI group, and adult studies were excluded from the present study.

**- Data extraction and risk of bias assessment**

The method of collecting and evaluating the data is described in detail in our previous meta-analyses (17-20). In summary, after searching and removing duplicates, two independent researchers reviewed the titles and abstracts of records and then full-texts of potentially eligible articles were assessed. Disagreements were resolved in consultation with a third reviewer. The data collection checklist was designed based on the PRISMA statement guidelines (21). Extracted data included first author's name, year of publication, sample size, age and sex distribution of patients, patients’ setting, AKI definition criteria, KIM-1 level assay method, time interval between patient’s admission and KIM-1 level assessment, the mean and standard deviation of urinary KIM-1 level, area under the receiver operating characteristic curve (AUC), and sensitivity and specificity of KIM-1 in predicting AKI in children. The risk of bias was assessed using the proposed guidelines in QUADAS-2: A Revised Tool for the Quality Assessment of Diagnostic Accuracy Studies (22).

**- Statistical analysis**

Data were recorded as mean and standard deviation, AUC, sensitivity, and specificity of KIM-1 in predicting AKI in children. Most studies reported median and interquartile range instead of mean and standard deviation. Therefore, Cochrane's proposed method was used to estimate the mean and standard deviation (23). All analyses were performed in STATA 14.0 statistical program and “*metan*” command was used. Findings were presented as standardized mean difference (SMD) with a 95% confidence interval (95% CI) to compare the mean urinary KIM-1 level in AKI group with non-AKI group. 

Since the time interval between admission and KIM-1 assessment varied between 0 and 96 hours in the included studies, analyses were performed in three time subgroups including 0-12 hours, 12-24 hours, and 24-96 hours. Also, in the eligible studies, patients were divided into three groups based on AKI severity, including stage 1 (or high-risk) AKI, stages 2-3 (or injury and failure) and all severities (stages 1-3). For this reason, the analysis was also performed based on these subgroups. For this purpose, being high risk was considered as stage 1 AKI, injury was deemed equivalent to stage 2 AKI, and failure was deemed equivalent to stage 3 AKI.

An additional analysis was performed to pool the AUCs reported for urinary KIM-1 level in predicting AKI in children. In this section, the AUCs of urinary KIM-1 with their 95% CI were recorded in the statistical program and an overall AUC was reported.

Heterogeneity between studies was assessed using I^2^ test and p value less than 0.1 were considered significant (indicating heterogeneity). In addition, publication bias was assessed using the funnel Plot (Egger's tests) (24).

## Results:

**- Characteristics of included studies**

**Figure 1:**
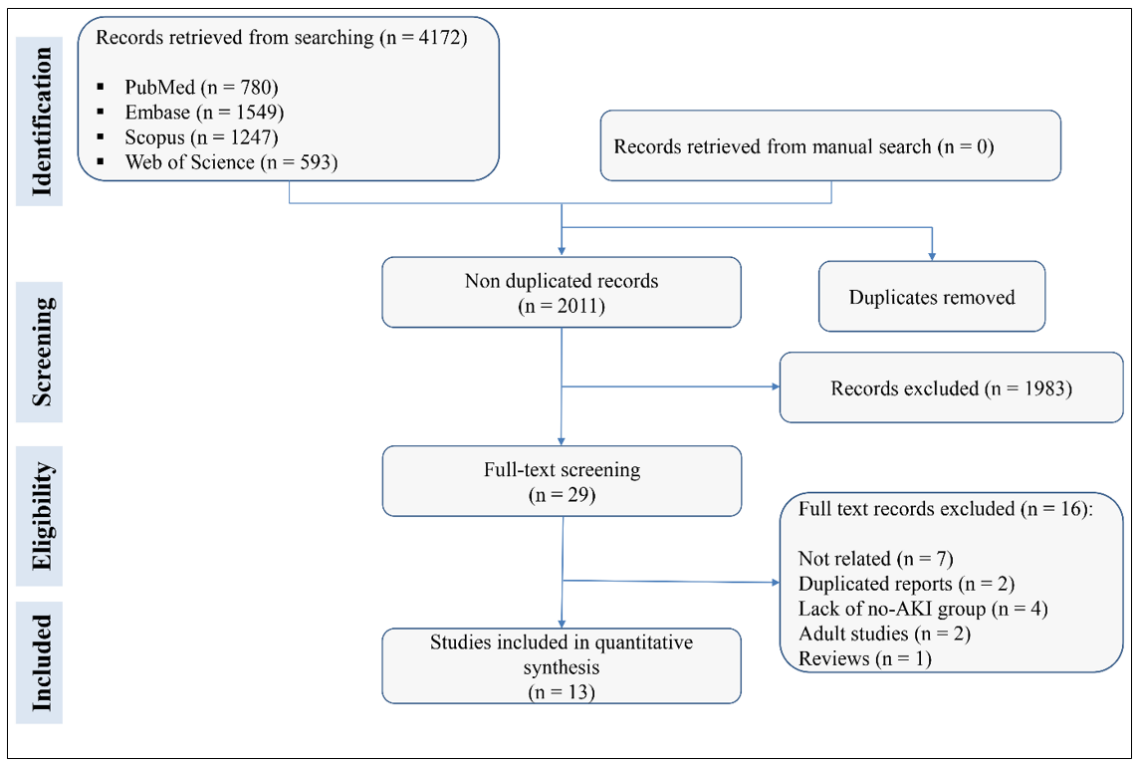
Flow diagram of screening and selection of eligible studies. AKI: Acute kidney injury.

A search of databases yielded 2011 non-duplicated studies. During screening, 29 articles were reviewed in detail, and finally the data of 13 articles were included in the present meta-analysis (25-37) (Figure 1). There were 8 cohort and 5 case-control studies. The sample sizes ranged from 33 to 252 children. The total sample size was 1620 children (825 of whom were boys). Identification of AKI in 8 studies were based on Kidney Disease Improvement Global Outcomes (KDIGO) criteria, based on Pediatric Risk, Injury, Failure, and End-stage kidney disease (pRIFLE) criteria in 4 studies and based on AKI network definition in 1 study. The interval between admission of patients and assessment of urinary KIM-1 levels ranged from 0 to 96 hours. All studies used the ELISA method to check the urine level of KIM-1 and all of them had frozen the specimens at -80 °C prior to examination. Table 1 shows the characteristics of the included studies.

**- Risk of bias and publication bias assessment**

**Figure 2:**
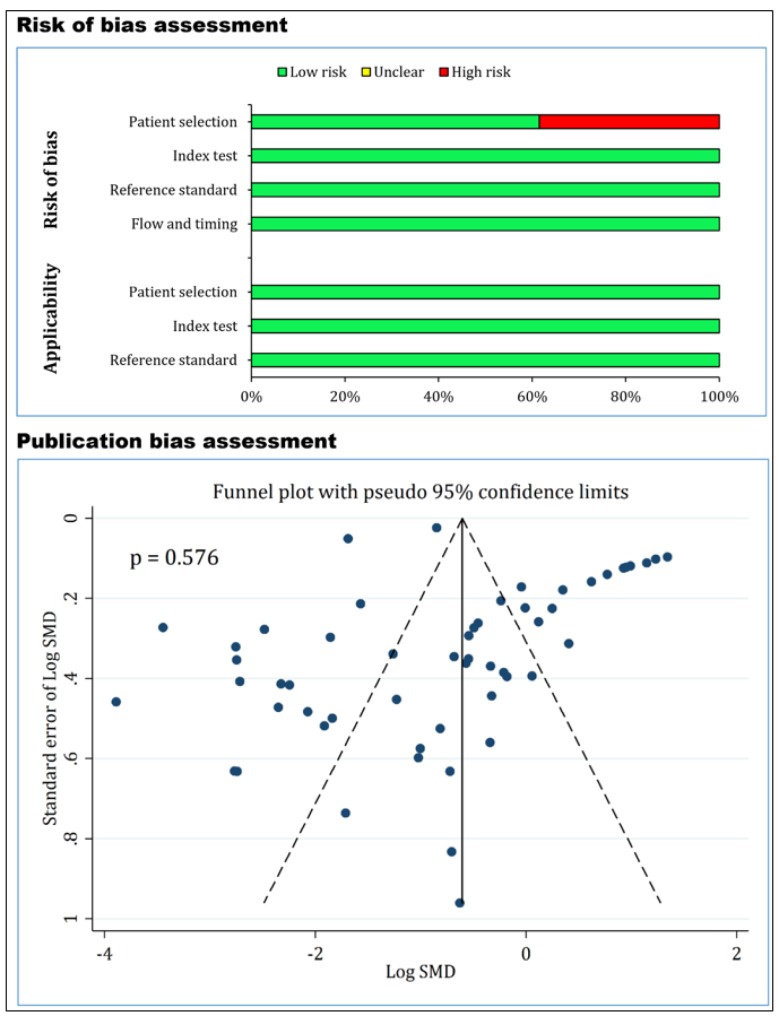
Risk of bias and publication bias assessment of the included studies. There is no evidence of publication bias (p = 0.576).

The quality control of studies was performed based on QUADAS-2 criteria. Since the design of 5 studies was case-control, patient selection was associated with bias in these 5 studies and therefore, they were marked as having high-risk of bias. In other cases, the risk of bias and applicability were low risk (Table 2 and Figure 2). The analysis also revealed no evidence of publication bias in the present study (p = 0.576) (Figure 2).

**- Comparison of mean urinary KIM-1 levels in children with and without AKI**

**Figure 3:**
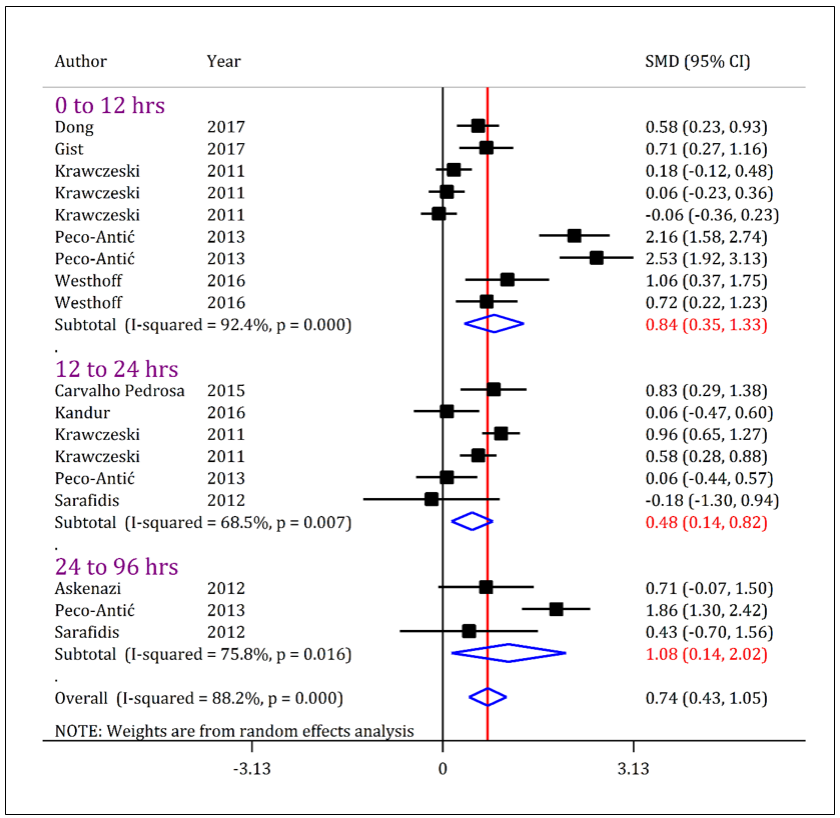
Forest plot for standardized mean difference (SMD) of urine kidney injury molecule-1 (KIM-1) between acute kidney injury (AKI) patients with all severities (stage 1/risk, stage 2/injury, and stage 3/failure) and non-AKI patients at different time cut offs. The urinary level of KIM-1 in AKI-patients is higher than non-AKI patients. CI: Confidence interval.

Urinary KIM-1 levels were significantly higher in children with AKI compared to non-AKI children, regardless of severity of AKI. As figure 3 shows, the urinary level of KIM-1 in children with all intensities of AKI (stage 1-3) was higher than non-AKI children during first 12 hours after admission (SMD = 0.84; 95% CI: 0.35 to 1.33; p = 0.001), 24-12 hours (SMD = 0.48; 95% CI: 0.14 to 0.82; p = 0.006) and between 96-24 hours (SMD = 1.08; 95% CI: 0.14 to 2.02; p = 0.024).

**- Comparison of mean urinary KIM1 levels between stage 1 AKI and non-AKI patients**

**Figure 4:**
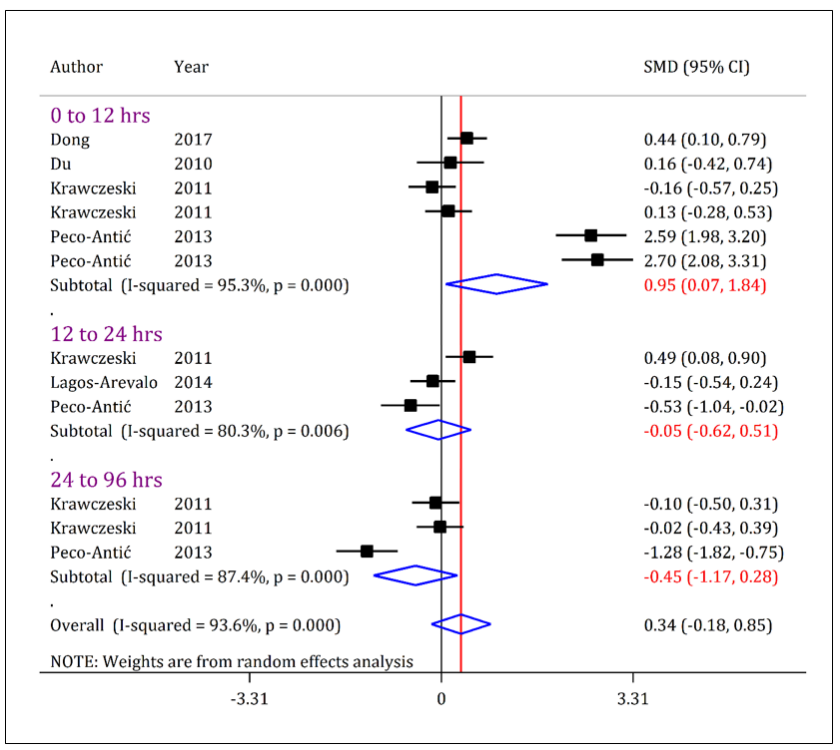
Forest plot for standardized mean difference (SMD) of urine kidney injury molecule-1 (KIM-1) between acute kidney injury (AKI) patients in stage 1/risk and non-AKI patients at different time cut offs. The urinary level of KIM-1 in AKI-patients with a severity of stage 1/risk is slightly higher than non-AKI patients only when assessed during the first 12-hours after admission. CI: Confidence interval.

The urinary level of KIM-1 in children with stage 1 AKI was higher than the non-AKI group only when examined within the first 12 hours of admission (SMD = 0.95; 95% CI: 0.07 to 1.84; p = 0.034). However, 12-24 hours (SMD = -0.05; 95% CI: -0.62 to 0.51; p = 0.859) and 24-96 hours (SMD = -0.45; 95% CI: -1.17 to 0.28; p = 0.226) after admission, there was no difference between stage 1 AKI and non-AKI groups (Figure 4).

**- Evaluation of mean urinary KIM-1 levels in stage 2-3 of AKI and non-AKI patients**

**Figure 5:**
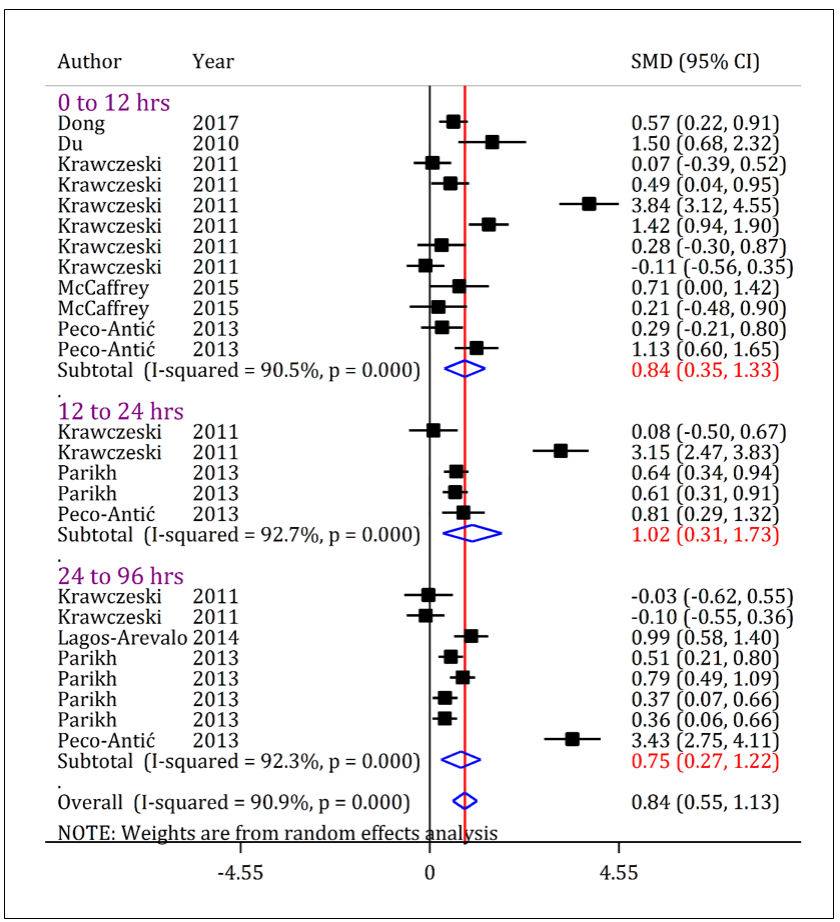
Forest plot for standardized mean difference (SMD) of urine kidney injury molecule-1 (KIM-1) between acute kidney injury (AKI) patients with stage 2-3/injury-failure severity and non-AKI patients at different time cut offs. The urinary level of KIM-1 in AKI-patients with a severity of stage 2-3/risk is higher than non-AKI patients in all assessed time points. CI: Confidence interval.

Urinary KIM-1 level in children with stage 2-3 AKI was significantly higher than non-AKI children. As figure 5 shows, the urinary KIM-1 level in children with stage 2-3 AKI were higher than non-AKI children within the first 12 hours (SMD = 0.84; 95% CI: 0.35 to 1.33; p = 0.001), 24-12 hours (SMD = 1.02; 95% CI: 0.31 to 1.73; p = 0.005) and 96-24 hours (SMD = 0.75; 95% CI: 0.27 to 1.22; p = 0.002) after admission.

**Discrimination**

**- The AUC of urinary KIM-1 level in diagnosis of pediatric AKI**

In four studies, AUC of urinary KIM-1 was reported with a 95% CI (26, 28, 33, 37). Pooled analysis showed that AUC of urinary KIM-1 in prediction of AKI was 0.69 (95% CI: 0.62 to 0.77).

**- Sensitivity and specificity of urinary KIM-1 level in diagnosis of pediatric acute kidney injury**

In the beginning of the present study, it was decided to evaluate the discriminatory power of urinary KIM-1 based on sensitivity, specificity and diagnostic odds ratio. To achieve this goal, we needed data of true positive, false positive, true negative and false negative. But such information was not reported in the studies.

Only three studies reported the sensitivity and specificity of urinary KIM-1 level in prediction of AKI. In the first study, Carvalho et al. showed that the best cut-off point for KIM-1 in predicting chemotherapy-induced AKI was 6.2 ng / mg of creatinine. In this cut-off, urinary KIM-1 had a sensitivity and specificity of 73.1% and 92.1%, respectively (26). Another study by Sarafidis et al. showed that based on the best cut-off point (cut-off = 569.8 pg / ml), KIM-1 had a sensitivity and specificity of 40% and 86%, respectively (36). Finally, Westhoff et al. reported sensitivity and specificity of 54.5% and 96.5%, respectively, for KIM-1; indicating that the best cut-off point for this urine biomarker was 2235 pg / ml (37).

## Discussion:

The findings of the present study showed that mean urinary KIM-1 level in children with stage 2-3 AKI was significantly higher than the non-AKI group. It was also found that the level of this biomarker in stage 1 AKI patients was only higher than the non-AKI group when assessed within the first 12 hours of admission. However, the AUC of urinary KIM-1 in prediction of AKI was 0.69, which is in the poor to fair range.

**Table 1:** Characteristics of the included studies

**Author; year; country**	**Study type**	**Setting**	**Age**	**Sample size**	**No. of boys**	**AKI definition**	**Timing (hrs)**
Askenazi; 2012; USA	Case-Control	AKI suspected	Neonates	33	17	AKI Network	0 to 96
Carvalho Pedrosa; 2015; Brazil	Cohort	Chemotherapy induced AKI	<18	64	26	KDIGO	24, 48, 72, 96
Dong; 2017; USA	Case-Control	Post-cardiopulmonary surgery AKI	<18	150	77	KDIGO	2, 6, 12
Du; 2010; USA	Cohort	AKI suspected	11.4	252	126	KDIGO	0
Gist; 2017; USA	Cohort	Post-cardiopulmonary surgery AKI	<1	94	63	KDIGO	6
Kandur; 2016; Turkey	Case-Control	ICU admitted AKI	1 to 17	60	33	KDIGO	24
Krawczeski; 2011; USA	Case-Control	Post-cardiopulmonary surgery AKI	<18	220	110	KDIGO	0, 6, 12, 24
Lagos-Arevalo; 2014; Canada	Cohort	AKI suspected	< 18	160	58	KDIGO	0 to 24
McCaffrey; 2015; UK	Cohort	AKI suspected	<18	49	26	pRIFLE criteria	0
Parikh; 2013; USA	Cohort	Post-cardiopulmonary surgery AKI	<18	311	171	pRIFLE criteria	6, 12, 24, 48, 72, 96
Peco-Antić; 2013; Serbia	Cohort	Post-cardiopulmonary surgery AKI	1.6	112	65	pRIFLE criteria	2, 6, 24, 48
Sarafidis; 2012; Greece	Case-Control	Asphyxia-associated AKI	Neonates	35	21	KDIGO	24, 72
Westhoff; 2016; Germany	Cohort	AKI suspected	<10	80	32	pRIFLE criteria	0

AKI: Acute kidney injury; KDIGO: Kidney Disease Improving Global Outcomes; pRIFLE: Pediatric Risk, Injury, Failure, Loss of kidney function, and End-stage kidney disease; Timing: Time interval between admission and kidney injury molecule-1 assessment.

**Table 2:** Quality assessment of included studies based on QUADAS-2 recommendations

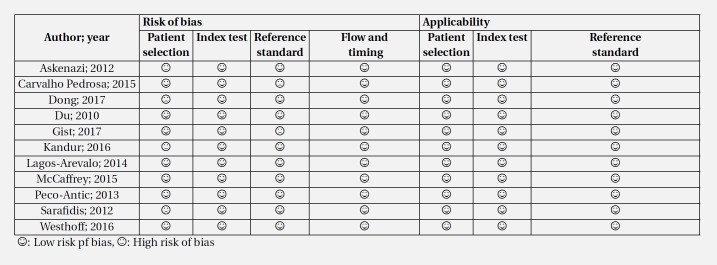

**Figure 6:**
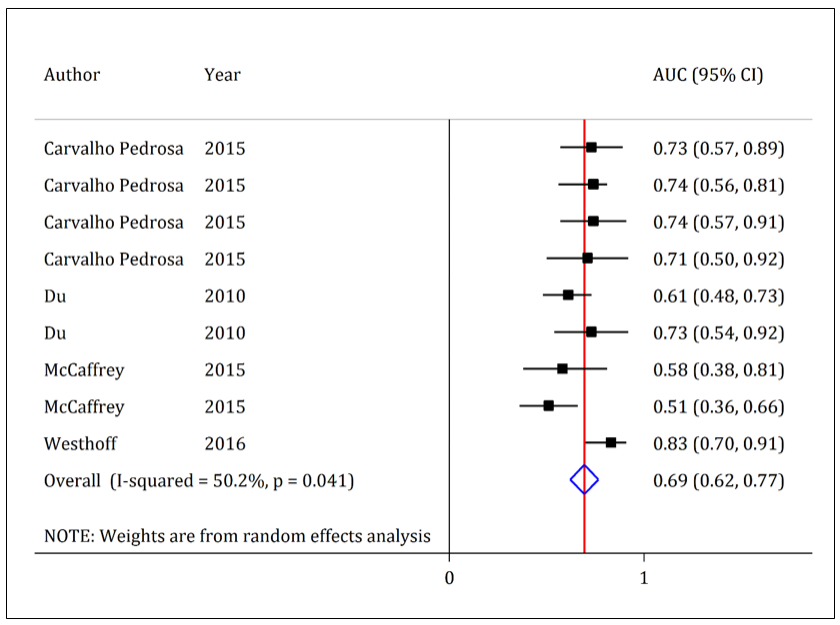
Area under the curve (AUC) of kidney injury molecule-1 (KIM-1) in diagnosis of acute kidney injury in children. The discriminatory power of KIM-1 in detection of acute kidney injury is poor to fair (AUC = 0.69; 95% confidence interval: 0.62 to 0.77).

Pooled analysis in the present study showed that the urinary level of KIM-1 was significantly higher in children with AKI compared to non-AKI cases. However, with a closer look at the findings, we will find that the obtained SMD is often below 1, which is in the poor to moderate effect size range. Therefore, KIM-1 may not be a good biomarker for the prediction of AKI in children. In addition, the AUC of this biomarker being poor to fair generally indicates that the discriminatory accuracy of KIM-1 is moderate at best.

Several methods have been suggested for assessing the discriminatory power of a biomarker. Although AUC calculation is the most common method in this field, it should be kept in mind that this test is a primary test and we require additional assessment such as sensitivity and specificity. However, sensitivity and specificity of KIM-1 were only reported in three studies. The sensitivity of KIM-1 to predict AKI in children was between 40% and 73.1% and its specificity was between 86% and 96.5%. This sensitivity and specificity were reported based on a wide range cut-off points. Therefore, we could not pool the data in this section. However, it seems that urinary KIM-1's sensitivity to predict pediatric AKI is poor to fair.

Other findings of the present study indicate the weakness of KIM-1 in differentiating patients at risk of AKI (stage 1 AKI) from non-AKI patients. It seems that the level of KIM-1 in the urine would increase significantly only when the patient is in the advanced stages of the AKI (injury or failure phase). This is a major limitation for KIM-1 in predicting AKI in children.

In a systematic review with the aim of examining the value of KIM-1 in diagnosis of AKI in children and adults, Wang et al. showed that the AUC, sensitivity and specificity of KIM-1 in prediction of AKI after cardiac surgery were 0.71, 76% and 0.84%, respectively (15). Although the findings of the study by Wang et al. are in line with the findings of the present study, there are major differences between the two studies. First, Wang et al.'s study pooled the findings of studies on adults and children; and second, out of the 15 included studies, only 3 were studies on children. Therefore, the findings of the study by Wang et al. could not be generalized to the pediatric community.

Along with KIM-1, there are other biomarkers such as cystatin C and NGAL that studies have cited as reliable indicators of AKI. Three previous meta-analyses showed that urinary and serum levels of cystatin C and NGAL had good to excellent discriminatory accuracy in predicting AKI. In the first study, Nakhjavan-Shahraki et al. showed that sensitivity and specificity of serum cystatin C in predicting AKI in children were 85% and 61%, respectively. The AUC of serum and urine cystatin C in predicting AKI were 0.83 and 0.85, respectively (4). In the other two meta-analyses, Izadi et al. showed that the serum level of NGAL in predicting AKI was 87% sensitive and 88% specific, and its urinary level had a sensitivity and specificity of 92% in predicting AKI. The AUC of serum and urinary NGAL in AKI prediction was 0.94 and 0.97, respectively (5, 6). Therefore, it seems that the use of NGAL and cystatin C biomarkers in predicting AKI is superior to KIM-1 in children.

**Limitations**

In the beginning of the present study, it was decided to calculate the overall sensitivity, specificity, and diagnostic odds ratio of KIM-1 in predicting AKI in children, but after searching and entering studies it became clear that such analysis was not possible. On the other hand, out of the 13 included studies, 5 were case-controls. In this type of design, the research team is aware of the existence of AKI in patients from the beginning, and this may lead to some degree of bias. Also, since a wide range of cut-off points were reported for KIM-1 in the studies, we were unable to reach a unique cut-off point for urinary KIM-1 in predicting AKI in children.

## Conclusion:

Based on available evidence, KIM-1 appears to have a moderate value in predicting childhood AKI. Since previous meta-analyses have shown urinary and serum biomarkers that have better discriminatory accuracy than KIM-1, it is better not to use urinary KIM-1 in predicting AKI in children.
